# *PRKCA* and Multiple Sclerosis: Association in Two Independent Populations

**DOI:** 10.1371/journal.pgen.0020042

**Published:** 2006-03-31

**Authors:** Janna Saarela, Suvi P Kallio, Daniel Chen, Alexandre Montpetit, Anne Jokiaho, Eva Choi, Rosanna Asselta, Denis Bronnikov, Matthew R Lincoln, A. Dessa Sadovnick, Pentti J Tienari, Keijo Koivisto, Aarno Palotie, George C Ebers, Thomas J Hudson, Leena Peltonen

**Affiliations:** 1 Department of Molecular Medicine, National Public Health Institute, Helsinki, Finland; 2 Research Program in Molecular Medicine at Biomedicum Helsinki, Helsinki, Finland; 3 Department of Human Genetics, David Geffen School of Medicine at UCLA, Los Angeles, California, United States of America; 4 McGill University and Genome Quebec Innovation Centre, Montreal, Quebec, Canada; 5 Department of Clinical Neurology, Radcliffe Infirmary, University of Oxford, Oxford, United Kingdom; 6 Department of Medical Genetics and Faculty of Medicine (Division of Neurology), University of British Columbia, Vancouver, British Columbia, Canada; 7 Department of Neurology, Helsinki University Central Hospital and Neuroscience Programme, Biomedicum, University of Helsinki, Helsinki, Finland; 8 Central Hospital of Seinäjoki, Seinäjoki, Finland; 9 The Broad Institute of MIT and Harvard, Cambridge, Massachusetts, United States of America; 10 Department of Clinical Chemistry, University of Helsinki, Helsinki, Finland; 11 The Finnish Genome Center, University of Helsinki, Helsinki, Finland; 12 Department of Laboratory Diagnostics, Helsinki University Central Hospital, Helsinki, Finland; 13 Department of Medical Genetics, University of Helsinki, Helsinki, Finland; University of Michigan, United States of America

## Abstract

Multiple sclerosis (MS) is a chronic disease of the central nervous system responsible for a large portion of neurological disabilities in young adults. Similar to what occurs in numerous complex diseases, both unknown environmental factors and genetic predisposition are required to generate MS. We ascertained a set of 63 Finnish MS families, originating from a high-risk region of the country, to identify a susceptibility gene within the previously established 3.4-Mb region on 17q24. Initial single nucleotide polymorphism (SNP)-based association implicated *PRKCA* (protein kinase C alpha) gene, and this association was replicated in an independent set of 148 Finnish MS families (*p* = 0.0004; remaining significant after correction for multiple testing). Further, a dense set of 211 SNPs evenly covering the *PRKCA* gene and the flanking regions was selected from the dbSNP database and analyzed in two large, independent MS cohorts: in 211 Finnish and 554 Canadian MS families. A multipoint SNP analysis indicated linkage to *PRKCA* and its telomeric flanking region in both populations, and SNP haplotype and genotype combination analyses revealed an allelic variant of *PRKCA,* which covers the region between introns 3 and 8, to be over-represented in Finnish MS cases (odds ratio = 1.34, 95% confidence interval 1.07–1.68). A second allelic variant, covering the same region of the *PRKCA* gene, showed somewhat stronger evidence for association in the Canadian families (odds ratio = 1.64, 95% confidence interval 1.39–1.94). Initial functional relevance for disease predisposition was suggested by the expression analysis: The transcript levels of *PRKCA* showed correlation with the copy number of the Finnish and Canadian “risk” haplotypes in CD4-negative mononuclear cells of five Finnish multiplex families and in lymphoblast cell lines of 11 Centre d'Etude du Polymorphisme Humain (CEPH) individuals of European origin.

## Synopsis

Complex diseases such as multiple sclerosis (MS) likely result from problems in networks of interactions between several genes and largely unidentified environmental and lifestyle factors. Identification of MS-specific genes has been challenging. HLA-DRB1*15 is the only consistent locus observed in most populations; however, the recent genome scan on more than 700 European families implicated 17q as a second-best MS locus [12]. Since MS families from the high-risk region of Finland initially revealed linkage to 17q, the authors used the regionally ascertained set of 63 families to identify a MS predisposing gene within a major non–HLA locus on 17q. The initial association was observed with single nucleotide polymorphisms (SNPs) located in intron 3 of the *PRKCA* (protein kinase C alpha) gene in Finnish MS families and replicated in an independent set of 148 MS families from Finland and 554 from Canada, two populations with a different genetic background. Combining the data of two SNP variants revealed two allele combinations of *PRKCA,* which were over-represented in Finnish or Canadian MS cases (odds ratio = 1.34, 95% confidence interval, 1.07–1.68, and odds ratio = 1.64, 95% confidence interval 1.39–1.94, respectively). Linkage and association of the *PRKCA* gene, encoding a regulator of immune responses, in two populations imply its involvement in the etiology of MS.

## Introduction

Multiple sclerosis (MS) is a chronic disease of the central nervous system responsible for a large portion of neurological disabilities in young adults. Similar to what occurs in numerous complex diseases, twin, adoption, and epidemiological studies indicate a complex etiology in which both unknown environmental factors and genetic predisposition are required to generate the disease [1–3]. Genome scans have revealed several putative susceptibility loci [4–7]. In addition to the human leukocyte antigen (HLA) locus on 6p, loci on 5p, 17q, and 19q have been replicated in multiple study samples [8–11]. Further, a recent high-density linkage screen utilizing 4,500 SNPs in 730 multiplex MS families of Northern European descent implicated the chromosome 17q as a locus with the second most significant maximum logarithm of odds (MLS) score (2.45) after the HLA [12].

The prevalence of MS in Finland is 50–100/10^5^, similar to other populations of Northern European descent living in a temperate climate [13]. However, in the Southern Ostrobothnian health-care district of Seinäjoki, located on the western coast of Finland, distinctly higher incidence (12/10^5^) and prevalence (200/10^5^) rates have been established [14,15]. This regional subisolate also shows exceptional familial clustering of MS [16]. Our genome-wide analyses have identified four main loci in Finnish MS families: the HLA class II region, the MBP locus on 18q, and two linked regions on 5p12-p14 and 17q22-q24 [7,17–19]. The relatively wide 17q locus, syntenic to rat experimental allergic encephalomyelitis *(Eae)* locus on rat chromosome 10 [20] was further restricted by haplotype analysis in Finnish families from the high-risk region to a 3.4-Mb region containing fewer than 20 transcripts [21]. The chromosomal architecture surrounding this critical MS locus was found to be complex, the area being flanked by large duplicated segments and areas enriched with palindromic sequence stretches, which are present also in the chimp genome [22]. Interestingly, the critical MS region is inverted in the chimp and human with respect to the order in the mouse genome.

To identify MS-associated allelic variants in Finnish families within this critical DNA region, we first constructed a sparse map of 67 SNP markers spanning the 3.4-Mb interval, and genotyped these SNPs in our primary material consisting of 22 multiplex and 41 trio families originating from the high-risk region. The same set of families was used in our previous fine-mapping study to define the critical DNA region on 17q [21]. The initial analysis revealed four SNPs of the *PRKCA* gene to show some evidence for association with MS [23]*.* To explore this gene and the flanking regions in more detail and to potentially identify specific allelic variant(s) predisposing to MS, we next constructed a denser panel of 211 SNPs covering and flanking the *PRKCA* gene, and genotyped them in two large, independent MS cohorts from Canada and Finland. The initial single SNP association was replicated in the second set of 148 Finnish families and an SNP haplotype analysis identified allelic variants of *PRKCA,* which were over-represented in Finnish (*n* = 211) and Canadian (*n* = 554) MS cases. Our initial association with *PRKCA* gene was also quickly reproduced in the United Kingdom case-control study [24]. This makes *PRKCA* a strong candidate for the gene underlying the linkage and association observed at this locus in several populations. Taking into account the critical role of the *PRKCA* and associated cellular pathways in regulating immune response, it may also be involved in other autoimmune diseases.

## Results

### Stage I: Preliminary Screen for SNP-Based Association on 17q24

**Figure 1:**
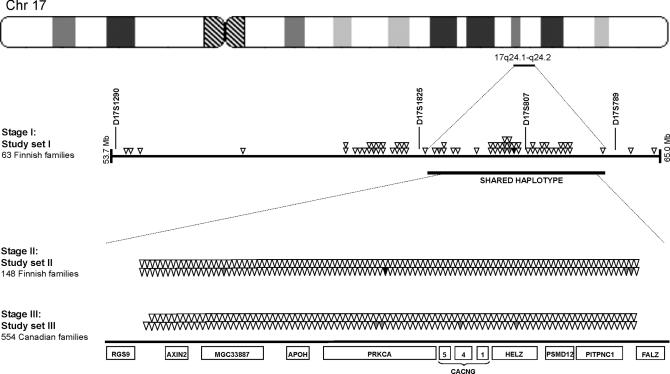
Schematic Representation of the MS Locus on Chromosome 17q24 Positions of the SNPs used in stages I, II, and III are shown with arrowheads. Grey arrowheads indicate SNPs showing evidence for association: stage I, *p* < 0.05; stage II, *p* < 0.01. SNP rs887797 showing evidence for association in the two independent Finnish study sets (Study sets I and II) is shown with a black arrow. The horizontal bar indicates the position of the critical MS region identified by haplotype analysis in our previous study [21]. The positions of the genes mapping to the locus are shown as boxes below.

Sixty-seven SNPs mapping to 53.7–65.0 Mb on 17q24, covering and flanking the critical 3.4-Mb MS region (Figure 1), were genotyped in a set of 22 multiplex and 41 trio families from the high-risk region containing a total of 327 individuals, including 97 MS cases (Table 1, Study set I). This regionally ascertained study sample should minimize the genetic and environmental heterogeneity. Forty of the 67 selected SNPs mapped within the 3.4-Mb DNA region translating to an average 85-kb inter-SNP interval. Nine SNPs showed some evidence for association to MS (*p* < 0.05) in transmission disequilibrium test (TDT) or haplotype-based haplotype relative risk (HHRR) analyses [25]. Strongest evidence for association was observed with a SNP rs7220007 (*p* = 0.002), which maps to the *PRKCA* gene. Three other SNPs of *PRKCA* also showed nominal evidence for association (rs759118, *p* = 0.02; rs741141, *p* = 0.01; and rs887797, *p* = 0.02). The minor allele frequencies and the results of TDT and HHRR analyses for all 67 SNPs are given in Table S1.

**Table 1:**
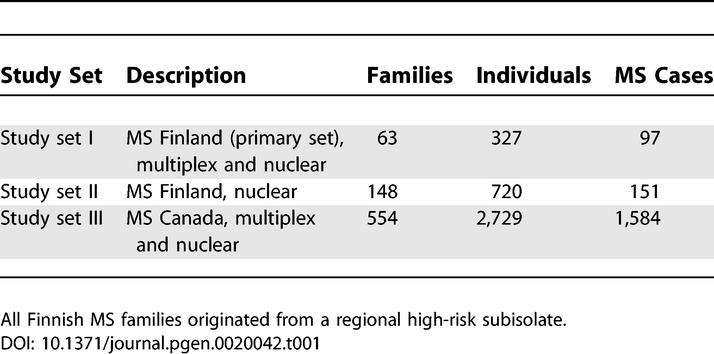
Description of the Study Material

### Stage II: Replication of the Association in an Independent Set of 148 Finnish MS Families

To capture most of the genetic variation of the *PRKCA* gene implicated as a primary positional candidate by the initial SNP-based analysis, and to potentially identify allelic variant(s) predisposing to MS, 211 SNPs mapping to the *PRKCA* gene and the 1-Mb flanking regions (see Materials and Methods for details) were selected from the dbSNP database (http://www.ncbi.nlm.nih.gov/projects/SNP) to evenly cover the region, and genotyped using a BeadArray platform in 211 Finnish MS families (Table 1, Study sets I + II). This provided a dense SNP map with an average 12-kb spacing between SNPs throughout the region, and an average inter-SNP distance of 8 kb within the *PRKCA* gene. Thirteen of the selected 211 SNPs had already been genotyped in 184 Finnish MS families originating from the high-risk region (as described for Stage I), and served here as controls between the different SNP genotyping platforms. We observed zero to one discrepancies for each SNP in the genotypes between the different genotyping platforms, making the discrepancy rate ≤ 0.15%.

A total of 148 of the 211 Finnish MS families (Table 1, Study set II) genotyped using the dense SNP set were independent from those utilized in stage I and were used here to replicate the initial SNP results. These families also originated from the high-risk regional subisolate, with a restricted number of founders. Four of the 178 high-quality SNPs with minor allele frequency ≥ 5% showed evidence for association in the HHRR analysis (*p* < 0.01) in this independent set of 148 Finnish MS families. The strongest evidence was observed with SNP rs887797 (*p* = 0.0004, the experiment-wide significance threshold to keep the type I error rate at 5% was 0.0005, thus the association remains significant after conservative correction for multiple testing), located in intron 3 of the *PRKCA* gene and implicated in the initial SNP analysis. The three other SNPs showing suggestive association were rs987931 (*p* = 0.009), rs1318 (*p* = 0.005), and rs2365403 (*p* = 0.003) (Figure 1 and Table 2). The HHRR results *p* < 0.05 are shown in Table 2, and the results for all 211 SNPs are given in Table S2. Table 2 also shows the HHRR results for the combined Finnish dataset (Study sets I + II). Association observed in the Finnish study sample seemed to be independent of the HLA, at least in this pre-selected set of families originating from a geographical high-risk region for MS (unpublished data).

**Table 2:**
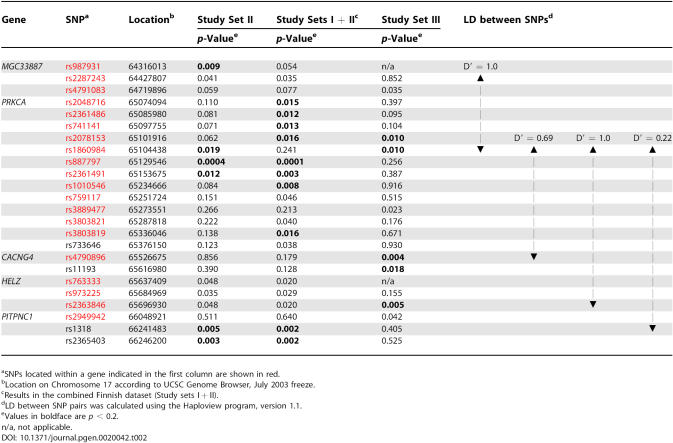
HHRR Association Results for the Dense Set of SNP (*p*-Values < 0.05) in Finnish and Canadian MS Families

### Stage III: Testing the Association of *PRKCA* Gene in a More Heterogeneous Canadian Population

To evaluate the relevance of the association observed in two independent sets of Finnish MS families in a more heterogeneous population, the same set of 211 SNPs was genotyped in 554 Canadian MS families (Table 1, Study set III) using a BeadArray platform. A total of 165 of the 211 SNP provided good quality data and had the minor allele frequency ≥ 5% in the Canadian sample (Table 2). Although the SNP showing strongest evidence for association in Finnish families, rs887797, failed to reveal association in Canadian families, two SNPs next to rs887797, located less than 28 kb apart, provided suggestive evidence for association in the 554 Canadian families (rs2078153, *p* = 0.0097, and rs1860984, *p* = 0.0099), the latter being also suggestively associated in the 148 Finnish families (*p* = 0.019) (Table 2). Two other SNPs with *p* < 0.01 in the Canadian MS sample were rs4790896 (*p* = 0.0038) and rs2363846 (*p* = 0.0046). The HHRR results *p* < 0.05 are shown in Table 2, and the results for all SNPs are given in Table S2.

To monitor for potential difference in background allele frequencies between two populations, we selected the ten most centromeric SNPs (rs724560–rs4630583, see Table S2), which did not show evidence for association to MS and were not in linkage disequilibrium (LD) with the SNPs of the *PRKCA* gene, and performed comparisons between Finns and Canadians using Genepop (Genepop on the Web [http://wbiomed.curtin.edu.au/genepop], option 3: population differentiation). A difference was observed in the distribution of genotypes between the Finnish and Canadian families (*p* = 0.026). Thus, the finding of different SNPs showing the strongest evidence for association in these two Caucasian populations is not totally surprising.

### Multipoint Linkage Analysis

The non-parametric linkage analysis option of MERLIN, which can accommodate marker-marker LD, was used to test for linkage in the Finnish and Canadian MS families [26]. Twelve SNPs covering *PRKCA* and the telomeric flanking region were selected for the multipoint analysis (for details see Materials and Methods). Two of the 12 SNPs (rs887797 and rs2078153) had pairwise *r^2^* above the selected threshold and were combined in the analysis. Although the information content of SNPs is lower than that of microsatellite markers, a maximum non-parametric linkage score of 2.33 in the combined dataset suggested evidence for linkage to the *PRKCA* region in both populations.

### LD Blocks and Haplotype Analyses of the *PRKCA* Locus

**Figure 2:**
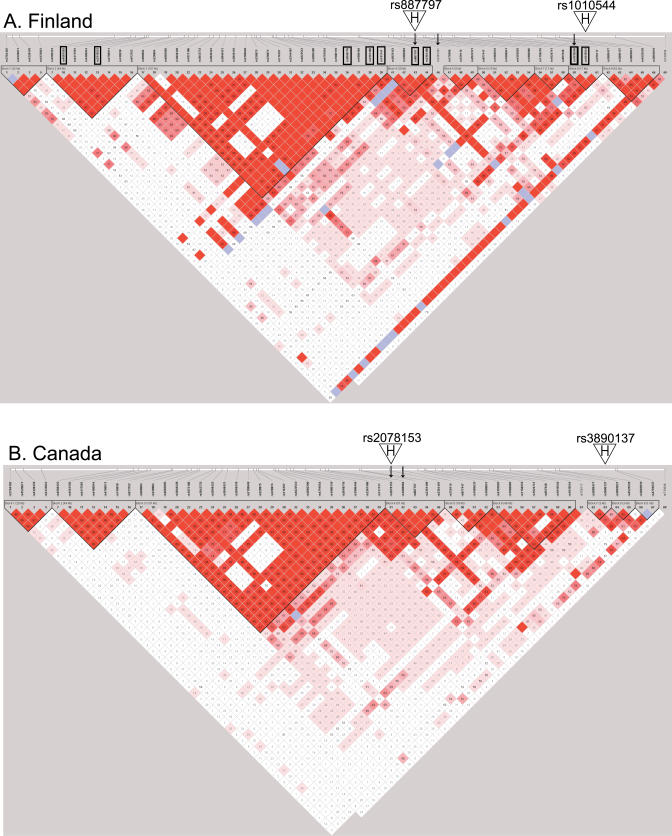
Haplotype Block Structure of the *PRKCA* Gene in Finnish and Canadian Populations (A) shows the block structures of the *PRKCA* gene (between SNPs rs3764402 and rs4791037) in 211 Finnish MS families; (B), in the 554 Canadian MS families. The haplotype blocks were created using the solid line of LD of the Haploview program, version 3.2 [27]. The SNPs providing strongest evidence for association to MS in *PRKCA* in Finns and Canadians (rs887797, rs2361491 and rs2078153, rs1860984, respectively) are marked with black arrows, SNPs used in Haploview version 3.2 [27] haplotype analysis in 211 Finnish families are indicated with boxes, and those used in the two SNP genotype combinations are indicated with a white arrowhead with the letter H and the rs-numbers given above.

To facilitate the analyses of allelic haplotypes, the level of LD within the *PRKCA* gene was evaluated using the Haploview program, version 3.2 [27]. We observed nine distinct haplotype blocks within the *PRKCA* gene, the size of which varied between 1 and 107 kb (Figure 2). Haplotype blocks 1–3 were identical in Finns and Canadians, but there were some differences in the block boundaries within blocks 4–9. The SNPs in blocks 4–9 show substantial LD with each other and with the SNPs in the 107-kb interval defined by block 3. The SNPs providing strongest evidence for association in the *PRKCA* gene in Finnish and Canadian MS families (rs887797 and rs2078153, respectively) are located in the haplotype block 4.

Because of the LD observed between SNPs located in blocks 3–9 (Figure 2), we decided to test also SNPs outside the block 4 in the haplotype analysis. Association was calculated for seven SNP haplotypes constructed from SNPs rs4561502, rs2319523, rs2048716, rs2361486, rs741141, rs887797, rs2361491, rs1010546, and rs1010544 (the positions of the SNPs are indicated in Figure 2) in 211 Finnish MS families using the TDT-based Haploview 3.2 custom association test option (results for all tested haplotypes are shown in Table S3) [27]. The C-A allele of the two-SNP haplotype (rs887797–rs1010544) was over-transmitted (corrected *p* = 0.039) and the T-A allele under-transmitted (corrected *p* = 0.031) to the MS cases of Finnish families. The same haplotype (rs887797–rs1010544) was also tested in the Canadian MS families, but no association was observed (*p* > 0.1). To test if there would be evidence for another predisposing allele in the Canadian population, we used SNP rs2078153, which shows the strongest evidence for association in Canadian families, and a second SNP, rs3890137, located in block 7, which is suggestively associated in Canadians, in the haplotype analysis (the positions of the SNPs are indicated in Figure 2); and observed the G-A allele to be under-transmitted (corrected *p* = 0.035) to MS patients in the Canadian families.

To extract all available information from the complete family sets, we also used genotype combinations of two SNPs (rs887797 and rs1010544 in Finns; rs2078153 and rs3890137 in Canadians) to monitor for potential allele frequency differences between the MS cases and the family controls, since only 30% of the Finnish and 32% of the Canadian MS families had both parents available, which is a requirement for a TDT-based analysis. The genotypes of one MS case (one random case from multiplex families) and one random healthy control from each family were used to calculate frequencies of observed allelic combinations of two-SNPs. The genotype combination analysis provided further evidence for these alleles defined by two-SNP haplotypes: The genotype combination (rs887797 and rs1010544) C-A was more frequent in Finnish MS cases compared to healthy controls from the same families (frequency: 0.58 in cases, 0.51 in controls, odds ratio [OR] = 1.34, 95% confidence interval [CI]: 1.07–1.68), whereas an allele combination T-A was under-represented in Finnish MS cases (Table 3). In the Canadian families, a genotype (rs2078153–rs3890137) combination C-A was over-represented in Canadian MS cases (frequency: 0.23 in cases, 0.15 in family controls, OR = 1.64, 95% CI, 1.39–1.94), whereas combination G-A was under-represented (Table 4).

**Table 3:**
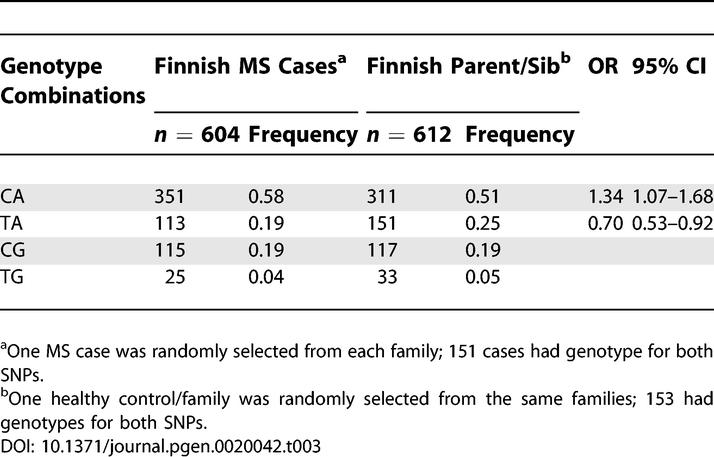
Two-SNP (rs887797 and rs1010544) Genotype Combinations in Finnish MS Families

**Table 4:**
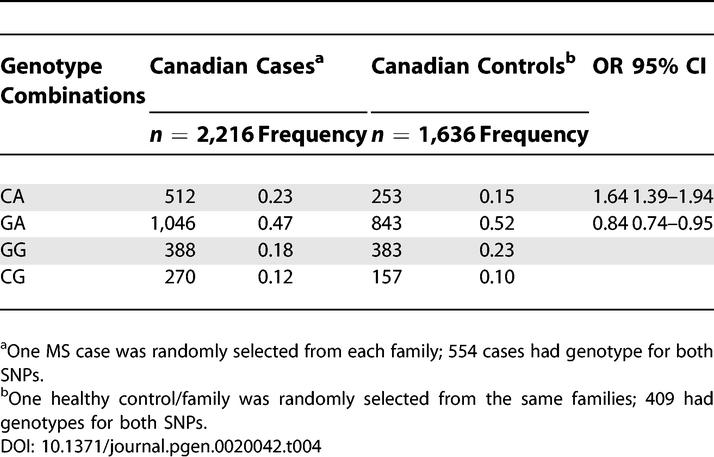
Two-SNP (rs2078153–rs3890137) Genotype Combinations in the Canadian MS Families

As another indication of the complexity of this genome region, we also saw putative evidence for association with the SNPs mapping telomeric to the *PITPNC1* gene in Finns, and these SNPs, unlike the suggestively associated SNPs in *CACNG* and *HELZ* genes, were not in high LD with the best *PRKCA* SNPs (see Table 2). To address potential involvement of this genome region, the Haploview 3.2 haplotype analysis was calculated for the two SNPs (rs2625563–rs1318), showing putative evidence for association as single SNPs in Finns, in Finnish and Canadian MS families. The two SNPs were in full LD and provided a *p*-value of 0.003 in Finnish families, but no evidence for association was observed in the Canadian families (*p* > 0.5).

### Sequencing of the Coding Regions of the *PRKCA* Gene

To exclude structural alterations in the *PRKCA* protein as a potential cause for MS, we sequenced the coding regions (including 30–50 bases of exon–intron boundaries) and 1.700 bases of the promoter of the *PRKCA* gene in ten unrelated Finnish MS patients, of which five had at least one copy of the putative disease allele (rs887797–rs1010544: C-A). We also sequenced the coding regions in eight healthy population controls. We found one polymorphic (GCC)-repeat located in the promoter region and 12 SNPs (Table 5). Three of the SNPs were located in the promoter region, two of them novel, whereas the other nine were either in exons or in exon–intron junctions (for details, see Table 5). SNP rs1010546, which showed nominal evidence for association in Finnish MS families, was located 11 bases from the junction of the exon 7 and in a sequence conserved between the mammalian species. However, we obtained no evidence of alternative splicing in blood mononuclear cell RNAs of four individuals (two cases and two controls), carrying the two different genotypes for this SNP (CC and CT).

**Table 5:**
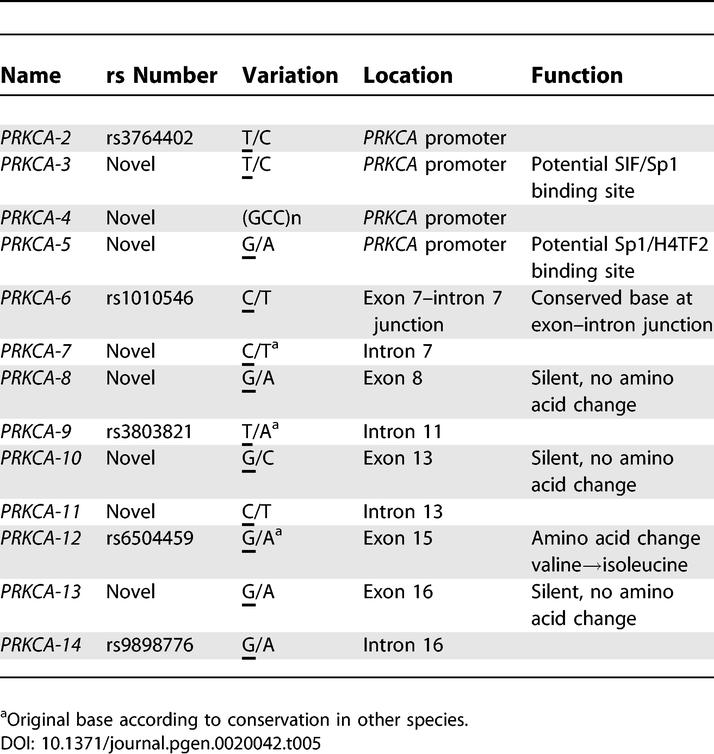
Sequencing of the Coding Regions of the *PRKCA* Gene

One of the novel SNPs, located in the exon 15, changes a codon of amino acid valine (GTC) to isoleucine (ATC), which seems to represent the ancestral amino acid in this position, the codon for isoleucine being conserved in the other species (chimp, mouse, rat, dog, chicken, fugu, and zebrafish). Although, the current human consensus sequence shows the allele G in this position, all sequenced healthy controls and five of the seven MS patients had an AA genotype in this position, whereas two MS patients had one or two copies of the G allele. These MS cases carried two and zero “risk” alleles of *PRKCA,* respectively. Further, we genotyped this G/A polymorphism in 30 Finnish nuclear MS families (96 individuals) and found the G allele to be rare (frequency 0.05) in the Finnish population: It was seen only in three of the 30 families. All individuals carrying the rare G-allele had at least one copy of the putative “risk” allele, but considering the low frequency of the G allele, this coding region variant cannot solely explain the association observed with the “risk” allele.

### Tissue and Cellular Expression of *PRKCA*

**Figure 3:**
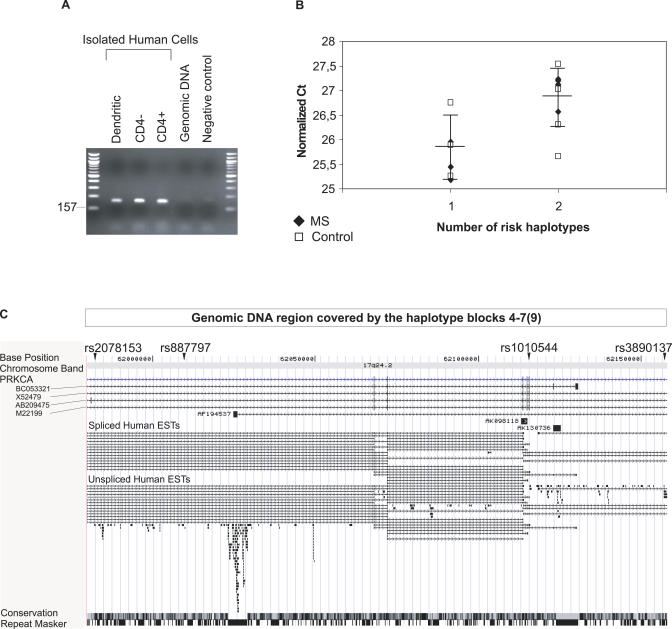
Expression of the *PRKCA* Gene and Overview of the Gene Region (A) RT-PCR analysis of expression of the *PRKCA* gene in isolated human cells. (B) Relative levels of the *PRKCA* gene expression in CD4^−^ blood mononuclear cells in individuals with one (*n*[controls] = 4, *n*[MS] = 3) or two copies (*n*[controls] = 4, *n*[MS] = 5) of the “risk” MS haplotype. Ct*(PRKCA)* values are normalized against mean of Ct*(HMBS)* and Ct(β-*actin*) and are shown on the *y*-axis and the number of putative disease haplotypes (rs887797–rs1010544: C-A) in the *x*-axis. (Ct = number of amplification cycles required to reach the selected signal threshold, thus higher Ct values mean lower relative expression levels). Expression in MS cases is shown with black diamonds and in healthy controls with white squares (average ± SD are given for all individuals as horizontal lines). (C) UCSC Human Genome Browser view of the critical region of the *PRKCA* gene (chr17:61,980,000–62,158,000). The genomic DNA region covered by haplotype blocks 4–7 (in Canadians) and blocks 4–9 (in Finns) is indicated on top. The ruler shows map positions on chromosome 17 (UCSC Genome Browser, April, 2004). Names and positions of the SNPs used in the haplotype analysis are shown above the ruler. The names of the mRNA clones corresponding to the *PRKCA* gene variants are shown on the left and to the three other mRNA clones beside them in the middle. The spliced and unspliced ESTs, respectively, are shown in the middle of the figure. Conservation between species and the repeat masker are indicated below.

To be relevant for the molecular pathogenesis of MS, a gene would be expected to be expressed either in tissues relevant for immune response, such as thymus or blood leukocytes, or in tissues affected by the disease process. Malfunctioning T lymphocytes or antigen-presenting cells could have a role in causing the disease. We studied the expression of the *PRKCA* gene by using the Human Fetal MTC and Human Immune System MTC cDNA panels (Clontech, Palo Alto, California, United States), and found it to be expressed in the relevant immunological tissues, like thymus, lymph node, and pool of blood leukocytes, as well as in a target tissue, the brain (unpublished data). RT-PCR revealed *PRKCA* mRNA to be expressed by antigen-presenting dendritic cells, by CD4^+^ T lymphocytes, and in a cell pool containing CD4^−^ blood mononuclear cells (Figure 3A).

We also monitored for the potential difference in the expression level of the *PRKCA* gene in the freshly collected peripheral blood mononuclear cells using quantitative real time RT-PCR. The cells were stratified to two cell populations according to the presence or absence of the CD4 surface antigen. The relative level of the *PRKCA* expression was measured in cells of MS cases and healthy members of five multiplex MS families (total of 20 samples). We compared the transcript levels in individuals with two or one “risk” alleles of *PRKCA* and observed an interesting difference: There was a 2-fold difference in the expression of *PRKCA* in CD4^−^ cells between individuals carrying one copy of the putative risk allele (*n* = 7) versus individuals who had two copies of the same allele (*n* = 9) (cycle threshold [Ct] = 25.9 ± 0.65 standard deviation [SD] and 26.9 ± 0.58 [SD], respectively), the expression being lower in individuals with two copies of the putative disease allele, disregarding the disease status (Figure 3B). The difference was somewhat larger in affected individuals (Ct = 25.5 ± 0.39 [SD] in heterozygotes and 27.0 ± 0.27 [SD] in homozygotes), which translates to 3-fold less *PRKCA* transcript in MS patients with two copies of the putative risk allele. This data suggests a regulatory element controlling the *PRKCA* expression segregating with the “risk” haplotype. Such a difference was not seen in CD4^+^ cells (0.7-fold difference, *n* =3 + 7, respectively). Samples from individuals without the putative risk allele were not available for the expression studies.

Due to non-accessibility of CD4-selected lymphocytes of Canadian patients, we addressed the role of the Canadian “risk” allele in the regulation of the *PRKCA* gene by using the publicly available expression and genotype data of Centre d'Etude du Polymorphisme Humain (CEPH) individuals from two recent publications by Monks and collaborators [28] and Hinds and co-workers [29]. Monks et al. [28] measured the expression of 23,499 genes in lymphoblastoid cell lines for members of 15 CEPH families. Hinds et al. characterized whole-genome patterns of human DNA variation by genotyping over 1.5 million SNPs in 71 CEPH individuals [29]. Eleven European-American CEPH individuals were utilized in both studies, and these samples had both expression and genotype data available for the *PRKCA* gene. We estimated the SNP haplotypes for the CEPH individuals of European ancestry using the Phase program (for details, see Materials and Methods) and found that one CEPH individual had two copies of the putative risk haplotype (rs2078153–3890137: C-A), one had one copy, and nine had no copies. Interestingly, similarly as observed for Finnish samples, the relative expression level of *PRKCA* was lower in the individual having two copies of the “risk” haplotype compared to individuals with one or no copies of that haplotype (0.004 versus 0.108).

### Other Potential Genes within *PRKCA*

Although the SNPs with the strongest association to MS are located in intron 3 of *PRKCA*, the analysis showed considerable inter-marker LD between most of the SNPs located in a DNA region covered by intron 3 through intron 8, making this more than 200-kb interval the most probable position for the disease susceptibility variant. According to the UCSC Human Genome Browser (May 2004 assembly; http://www.genome.ucsc.edu), there are two variant forms of *PRKCA:* mRNA variant AB209475 includes an additional exon in the intron 3, and the other variant, BC053321, two exons in the intron 8 (Figure 3C). The first variant form was observed in a brain library, but there are no EST (expressed sequence tag) clones corresponding to the variant exon 3b. The second variant mRNA was identified in an epidermoid carcinoma library, and an EST clone BM020079 containing the same sequences existed in an astrocytoma library. Further, there are three other mRNAs with unknown function mapping to this interval: AF194537, AK098118, and AK130736. We also made an effort to identify all the EST clones mapping to the critical interval using the UCSC Human Genome Browser (Figure 3C). We could identify over 120 non-overlapping EST clones within this DNA region, over 70 within intron 3.

## Discussion

Complex diseases such as MS most probably result from problems in networks of interactions between several genes and as yet unidentified environmental and lifestyle factors. Even though studies in MS families have identified a limited number of linked loci, replicated in several populations, identification of MS genes or DNA variants specific to MS has been a challenging task, and several initial findings showing association, especially to candidate genes, have failed to be replicated in other populations. Only association of MS with HLA-DRB1*15 has been confirmed in most studies (reviewed in [30]), yet the role of this locus in MS susceptibility has remained unknown. Here we aimed to identify an MS gene and disease-associated allelic variants at the 17q locus for which we have established linkage and initial evidence for a restricted chromosomal region among individuals with MS in Finnish families originating from a high-risk subisolate of Finland [21]. A recent high-density SNP linkage scan indicated this locus as a locus with the second highest MLS score in a large collection of families of Northern European descent [12]. Initial evidence for association with SNPs mapping to intron 3 of the *PRKCA* gene was established in the same Finnish study samples providing evidence for linkage. This association with *PRKCA* was replicated in the second set of 148 MS nuclear families collected from the high-risk region. Considering the historical and demographic differences between Finns and Canadians, as well as the difference in the background allele frequencies between these two populations, it was not surprising that the MS-associated allelic haplotypes were defined by different sets of SNPs. A comparable difference was observed for SNPs within the HLA region between these same sets of MS families: Different SNP blocks within the HLA class II region showed strongest association to MS in Finnish and Canadian families [19]. However, both the Finnish (rs887797–rs1010544: C-A) and the Canadian (rs2078153–rs3890137: C-A) risk haplotype covers the same region of the *PRCKA* gene: a DNA segment from intron 3 through intron 8. The observed ORs for both populations are in good agreement with the λ_s_ value of 1.18 estimated for this locus by the International Multiple Sclerosis Consortium linkage screen [12].

A recent study in the UK population [24] replicated our initial finding of the *PRKCA* association [23]. A total of 35 SNPs mapping to *PRKCA* were genotyped in 184 unrelated MS cases and 340 controls collected from the UK. Three SNPs showing putative evidence for association (best *p*-value = 0.0018) were located in the 3′ end of *PRKCA,* whereas the two associated two-SNP haplotypes covered the first two exons and the beginning of intron 2 (PRKCA*1_2) and the last intron and exon of *PRKCA* (PRKCA*30_31), thus flanking the genetic region showing association in Finns and Canadians.

In addition to the *PRKCA* gene, we observed some evidence for association to MS with individual SNPs located in *CACNG/HELZ* genes in Canadian MS families and telomeric to the *PITPNC1* gene in Finnish MS families. This can be explained by LD between the best *PRKCA* SNPs and the SNPs in *CACNG/HELZ* genes. However, no LD was found between the critical *PRKCA* SNPs and the *PITPNC1* SNPs, thus this feature cannot explain the observed association on this region. The Canadian study sample did not show any association with *PITPNC1* SNPs (rs1318 and rs2365403), which tempers the interest to this region. However, considering the complex structure of this chromosomal region, the more complex association pattern on 17q could also indicate that the SNPs in the broader region showing association to MS are in linkage disequilibrium with an as yet unidentified duplication, inversion, or another chromosomal rearrangement polymorphism affecting some or several genes located within this interval. A recent study by Gonzalez and co-workers [31] observed significant interindividual and interpopulation differences in a copy number of a segmental duplication encircled gene encoding *CCL3L1* and found that possession of a *CCL3L1* copy number lower than the population average markedly enhanced HIV susceptibility. Stefansson and co-workers [32] also recently identified a common inversion, which is under positive selection in Europeans and maps to 17q21.31. We have shown that the critical MS region on 17q24 is flanked by palindromic segments and highly-homologous duplicated sequences, which can predispose to large chromosomal rearrangements by nonallelic homologous recombination [22].

*PRKCA* is an excellent functional candidate for predisposition to MS, since the disease is believed to be the result of misdirected immune response by autoreactive T cells against myelin antigens [33], and protein kinase C (PKC) plays a critical role in signal transduction controlling T-lymphocyte activation. The *PRKCA* has been functionally implicated in T-cell receptor/CD28-induced interleukin 2 *(IL-2)* gene expression and has also a critical role in regulating *IL-2* receptor expression (reviewed in [34]). *IL-2* can initiate cell cycle progression during T-cell activation. *IL-2* signals via the *IL-2* receptor and phosphatidylinositol 3-kinase *(PI3K),* a pathway mediated downstream by the *E2F* transcription factor family [35]. A recent study by Iglesias and co-workers [36] observed enhanced *E2F* pathway transcription in peripheral blood mononuclear cells from MS patients. We can hypothesize that the enhanced transcription of *E2F* pathway genes could be a result of altered *PRKCA* activity in those patients.

We observed a distinct difference in the relative levels of the *PRKCA* expression in CD4^−^ mononuclear cells between individuals with one or two copies of the risk haplotype identified here, regardless of the disease status. Interestingly, expression and genotype data available in the public databases supported our findings: The *PRKCA* expression seemed to correlate with the copy number of the Canadian risk allele in lymphoblast cell lines derived from the CEPH individuals of European ancestry. We acknowledge that the number of samples is still too small for definitive conclusions, but further studies to evaluate whether a variant segregating with the putative disease haplotype has an effect in the expression of the *PRKCA* are warranted. Also, the blood mononuclear cell pool might not be the optimal cell type to look for the potential difference in the expression of the *PRKCA* gene; this feature should also be assessed in the antigen-presenting cells.

The *MBP* locus on chromosome 18q is another locus showing evidence of linkage and association to MS in these Finnish families [17]. Interestingly, Feng and co-workers [37] showed that a *golli* product of the myelin basic protein *(MBP)* gene can serve as a negative regulator of signaling pathways in T lymphocytes, particularly involving the PKC pathway. The *golli* product expressed in T lymphocytes, BG21, can be phosphorylated by *PKC,* but the inhibition of the PKC pathway is independent of the phosphorylation. The negative regulation by BG21 is not due to direct action on *PKC* activation, but in the cascade following *PKC* activation. Thus these two loci may actually act in concert to confer susceptibility to MS.

Considering the role of the *PRKCA* in regulating immune response of T cells and the fact that this locus on chromosome 17q was found to be one of the human non-HLA autoimmune clusters sharing conserved genetic location with animal autoimmune loci [38], it is tempting to speculate that *PRKCA* might also have a role in susceptibility to other autoimmune diseases, especially those mapping to the 17q24 locus, like rheumatoid arthritis and psoriasis. Barton and co-workers [39] observed both linkage to the region and association to a marker D17S807, which is located next to the *PRKCA* gene, in rheumatoid arthritis ASP families, when studying human loci syntenic to two rat models of inflammatory arthritis. A genome-wide scan by Nair and collaborators [40] provided evidence for a psoriasis susceptibility locus also on this region of 17q. The *PRKCA* gene is one of the key components in a pathway that regulates immune responses, and is located in a region shown to harbor susceptibility gene(s) for several human autoimmune diseases and animal autoimmune/inflammatory disease models. Thus it serves as an excellent functional and positional candidate for predisposition to other human and animal autoimmune diseases.

## Materials and Methods

### Finnish MS pedigrees.

The primary study material consisted of 22 multiplex MS families with two to six affected cases per pedigree, and 41 MS patients with their parents and/or unaffected sibs, all originating from the high-risk region for MS in the Southern Ostrobothnia of Finland (Table 1, Study set I). The families are identical to those previously described in our fine-mapping study [21]. In an extension to the primary study sample, 148 nuclear families (affected family member and her/his parents or, in case of a missing parent, two to four healthy siblings) have been collected from the same region in Southern Ostrobothnia (Table 1, Study set II). There were altogether 63 full trios (MS case + both parents) available in these datasets. All families were Finnish and of Caucasian descent, and at least one of the parents was born in Southern Ostrobothnia. Diagnosis of MS in affected individuals strictly followed Poser's diagnostic criteria [41]. All individuals gave their informed consent, and the study has been approved by the Ethics Committee for Ophthalmology, Otorhinolaryngology, Neurology, and Neurosurgery in the Hospital District of Helsinki and Uusimaa. (Decision 46/2002, Dnro 192/E9/02).

### Canadian MS pedigrees.

A cohort of 554 MS families were ascertained as part of the ongoing Canadian Collaborative Project on the Genetic Susceptibility to MS (CCPGSMS), for which the methodology has been described previously [42]. This cohort consists of families with at least one affected sibling pair (*n* = 399), families with affected parent-child pairs (*n* = 49), simplex (*n* = 97), and extended families (*n* = 21). There were 211 full trios (MS case + both parents) available in this dataset. All families were Canadian and of Caucasian descent.

### Genotyping.

To control for sample mix-ups, all Finnish samples were analyzed to determine the gender of the sample and genotypes of four microsatellite markers using the ABI 3730 (Applied Biosystems, Foster City, California, United States). The data were compared to the known sex of the samples and checked for Mendelian errors. Discrepant samples were excluded from the analyses.

Stage I: We identified a set of 135 SNPs mapping to 53.7–65.0 Mb on 17q24 between markers D17S1290 and D17S789 on chromosome 17, covering and flanking the 3.4-Mb MS region, which showed evidence for excessive allele sharing and linkage to MS in large Finnish multiplex families (Figure 1). At the time (1999), a significant proportion of the SNPs in the publicly available database were not verified, thus only 67 of 135 SNPs were proven to be polymorphic in our sample set and passed our quality criteria for large-scale genotyping. SNPs in stage I were genotyped using the MassARRAY System (Sequenom, San Diego, California, United States), allele-specific primer extension on microarray or Pyrosequencing (Pyrosequencing, Uppsala, Sweden). Details for individual SNPs provided upon request from JS. For the MassARRAY System (Sequenom), we used 5 ng of DNA/reaction. The SNP assay was designed using SpectroDESIGNER (Sequenom), and the PCR and extension reactions were done as specified by the manufacturer. Genotypes were automatically called with the SpectroCALLER software (Sequenom), and manually checked as described [43]. Part of the SNPs in stage I were genotyped in a multiplex using allele-specific primer extension on microarrays with a protocol developed in our laboratory and described by Pastinen and co-workers [44] with minor modifications. Two allele-specific detection oligonucleotides were designed for each SNP and synthesized (Operon, Alameda, California, United States) with a 5′aminolinker and a poly-T (9T) stretch followed by the SNP-specific sequence. The oligonucleotides were spotted in duplicate onto silane/isothiocyanate-coated chrome mirror microscope slides (Evaporated Coatings, Willow Grove, Pennsylvania, United States) using a robot built in-house. Sequences containing the SNPs were amplified from genomic DNA in multiplex PCR reactions (up to five SNPs together) using SNP-specific primers with T3 (forward) or T7 (reverse) tails. (Specific PCR conditions can be obtained from author JS by request). Multiplex reactions were pooled and in vitro transcribed using the AmpliScribe T7 or T3 High Yield Trancsription Kit (Epicentre Technologies, Madison, Wisconsin, United States). The DNase I–treated T3 or T7 RNA pools were hybridized to the arrays in a hybridization buffer containing 1.67 M NaCl at 42 °C for 20 min. Arrays were washed twice in 0.3M NaCl, 0.5 × TE, 0.1% TritonX-100, followed by a short rinse in ice-cold water. Subsequently, the allele-specific extension was performed using MMLV Reverse Transcriptase (Epicentre Technologies) in a 20-μl reaction containing 50 mM Tris-HCl (pH 8.3), 10 mM MgCl_2_, 75 mM KCl, 10 mM DTT, 0.5 μM dATP, 0.5 μM dGTP, 0.5 μM ddATP, 0.5 μM ddGTP, 1.0 μM FluoroLink Cy5-dCTP, 1 μM FluoroLink Cy5-dUTP (both Amersham Pharmacia Biotech, Piscataway, New Jersey, United States), 15% glycerol, and 0.24 M Trehalose, at 50 °C for 20 min. A 5′ Cy3-(A)_9_ 3′ blocked probe (Operon) was added to the hybridization reaction to monitor for efficiency in the array production. Finally, arrays were washed twice in 0.3M NaCl, 0.5 × TE, 0.1% TritonX-100, followed by a short rinse in ice-cold water, air dried, and scanned using a GMS418 Array Scanner (Affymetrix, Santa Clara, California, United States). The image was analyzed using the Scanalyze software (http//:rana.lbl.gov/EisenSoftware.htm). Genotypes were clustered and called using a program developed in-house (W. Wong and C. Li, unpublished data).

The rest of the SNPs analyzed in stage I were genotyped using Pyrosequencing as specified by the manufacturer.

Stage II: 211 SNPs mapping to the *PRKCA* gene and to 1-Mb centromeric and telomeric flanking regions (chr17: 60.72–63.23 Mb) were identified from the dbSNP database and selected to evenly cover the *PRKCA* gene and its 1-Mb flanking regions. The SNP selection was independent of stage I, except that the SNPs (rs759118, rs741141, rs7220007, and rs887797) showing evidence for association to MS in stage I were also included here. Selected SNPs were genotyped using the BeadArray platform (Illumina, San Diego, California, United States) as described in Fan and co-workers [45]. Thirteen SNPs already genotyped in stage I were re-genotyped by BeadArray platform in order to evaluate the accuracy of the genotyping. We found zero to one discrepancies/676 genotyped individuals for each SNP between the platforms, making the discrepancy rate less than 0.15%. All SNP genotypes were checked for Mendelian errors. Of the 211 SNPs, 178 and 165 markers had minor allele frequency greater than 5% and genotype call rate greater than 90% in the Finnish and Canadian samples, respectively, and were used in the statistical analyses.

### Statistical analyses.

SNP genotypes obtained from different genotyping platforms were coded as follows: A = 1, C = 2, G = 3, and T = 4. Allele calling depended on the orientation from which the SNP was genotyped. Allele and genotype frequencies were determined from the data, and deviation from the Hardy-Weinberg equilibrium was tested using Pearson's chi-square test. HHRR and TDT analyses of the ANALYZE package were used in the primary analysis (stage I) to monitor for association [25]. TDT and HHRR utilize only the data of full trios (MS case + both parents, one trio is utilized in families with multiple MS cases), but HHRR is able to make use of families in which both parents are not heterozygotes for a given marker. Thus HHRR was used in the stage II analysis. We used the SNPSpD method (http://genepi.qimr.edu.au/general/daleN/SNPSpD) [46] with modifications by Li and Ji [47] to correct for multiple testing. According to SNPSpD, the effective number of independent SNP loci was 100.1808, thus the experiment-wide significance threshold of 0.0005 was required to keep the type I error rate at 5%. In the text, all *p*-values for individual SNPs are presented as non-corrected.

To test for potential interaction between the HLA locus and the locus on chromosome 17q24, we stratified the Finnish MS families first according to the HLA type using SNP rs2239802, which is located in the HLA region and shows strong evidence for association (*p* = 3 × 10^−10^ in HHRR) to MS. Families were divided into three subgroups based on whether the affected individual had two, one, or no copies of the risk SNP allele (*n* = 46, *n* = 76, and *n* = 48 families/subgroup, respectively), and association was monitored in each subgroup using the HHRR.

Genepop Option 3, sub-option 3 (population differentiation: genotypic differentiation; http://wbiomed.curtin.edu.au/genepop) was used to compare the background allele frequencies between Finnish and Canadian MS families. The genotypes of the ten most centromeric SNPs (Table S2, rs724560–rs4630583) of 265 independent, healthy Finnish and Canadian individuals were utilized in the analysis with the default Markov chain parameters. A corrected *p*-value is given in the text (*df* = 20, Fisher's method).

We used the non-parametric multipoint linkage analysis of MERLIN (version 0.10.2, http://www.sph.umich.edu/csg/abecasis/Merlin/index.html) to test for evidence of linkage in the combined set of Finnish and Canadian families, since it can accommodate LD between markers [26]. MERLIN's option rsq *q (q=0.01)*, which calculates pairwise *r^2^* between neighboring markers and creates a cluster joining markers for which pairwise *r^2^* > *q* and all intervening markers, was employed. Twelve SNPs were selected for the multipoint analysis: one SNP from haplotype blocks 1–3 of the *PRKCA* gene (rs3764402, rs973752, rs2361486), two strongest associating SNPs (rs2078153, rs887797) from block 4, rs1985633 from blocks 5–6, and rs3889477 from block 9 (Finnish)/blocks 7–9 (Canadian). In addition to the *PRKCA* SNPs, we included SNPs rs733646 (telomeric to *PRKCA*), rs4790896 *(CACNG4),* rs2363846 *(HELZ),* rs2949942 *(PITPNC1),* and rs2365403 *(AK094724)*.

The level of LD between the SNPs was calculated using all polymorphic SNPs located in *PRKCA* in Finnish and in Canadian MS trios with the Haploview program, version 3.2 [27]. The Haploview 3.2 custom association test option was used to monitor for association of seven haplotypes formed by SNPs rs4561502, rs2319523, rs2048716, rs2361486, rs741141, rs887797, rs2361491, rs1010546, and rs1010544 (indicated by boxes in Figure 2), which either showed association in 211 Finnish MS families as single SNPs or were located in the same haplotype block as an associated SNP. The haplotype showing strongest association in Finns (rs887797–rs1010544) was tested in Canadian families using the Haploview 3.2. custom test option, as was a haplotype of SNPs rs2078153 and rs3890137, which showed putative association as single SNPs or were in the same block as a putatively associated SNP in Canadian MS families. Haploview 3.2 was also used to calculate haplotype association for SNPs rs2625563–rs1318, covering the DNA region telomeric to the *PITPNC1* gene, in Finnish and Canadian families. *p*-Values for putative risk/protective haplotypes (rs887797–rs1010544:C-A/T-A in Finnish and rs2078153–rs3890137:G-A in Canadian MS families) were calculated by comparing one allele to the other three alleles, and the *p*-values were corrected for multiple testing using the permutation option available in Haploview 3.2 (1,000 permutations).

Since only one third of the families had both parents available, we further used genotype combinations to test for differences between the MS cases and family controls in the Finnish and Canadian datasets. One MS case (one randomly selected MS case from multicase families) and one random control individual were selected from each family to calculate the observed genotype combinations for SNPs rs887797 and rs1010544 in Finns and SNPs rs2078153 and rs3890137 in Canadians. ORs were calculated by comparing the putative risk/protective allele to all the other alleles.

Individual two-SNP haplotypes (rs887797–rs1010544) were assigned for members of the multiplex families used in the *PRKCA* expression analysis utilizing the sufficient phase information available in these families.

### Sequencing.

MS cases of trio families and one MS patient from multiplex families were randomly selected for sequencing. Anonymous Finnish control DNAs were used as reference samples. The primers were designed by using the on-line primer design software Primer3 input (http://frodo.wi.mit.edu/cgi-bin/primer3/primer3_www.cgi). All primers had a melting temperature (Tm) between 58 °C and 60 °C. The segment was amplified by PCR and sequenced in both directions using the ABI PRISM 377 DNA Sequencer (Applied Biosystems) with BigDye terminator chemistry and standard conditions (Applied Biosystems). The sequence data was analyzed using ABI Sequencing Analysis 3.3 (Applied Biosystems), Sequencher 4.0.5 (Gene Codes Corp., Ann Arbor, Michigan, United States), and the published genome sequence (UCSC Human Genome Browser, July 2003 freeze, http://www.genome.ucsc.edu).

### Alternative splicing analysis.

Alternative splicing analysis was carried out on 1 μl of cDNA prepared from CD4^+^ and CD4^−^ cell populations from four individuals (two cases and two controls) as described below. Both alleles of the rs1010546 SNP were PCR-amplified under standard conditions with the use of an exonic primer couple (amplifying a product spanning from exon 6 to exon 8; primer sequences are available on request).

### MS and control lymphocyte samples.

We collected fresh blood samples from 24 members of five extended MS families. Samples were obtained from MS cases and healthy family members, of whom 13 were females and 11 were males. All donors gave their informed consent, and the study was approved by ethical committees of Helsinki University and Seinäjoki Central Hospitals.

The lymphocytes were immediately harvested using Ficoll-Paque density gradient centrifugation and further stratified to CD4^+^ and CD4^−^ cell populations by negative selection with CD4^+^ T cell isolation kit (Miltenyi Biotec, Auburn, California, United States) using autoMACS instrument (Miltenyi Biotec). The total cellular RNA was extracted using Trizol reagent followed by DNAse I treatment and additional purification with RNeasy Mini Kit columns (Qiagen, Valencia, California, United States) according to the manufacturers' instruction. The quality of the RNA was assessed using the RNA 6000 Nano assay in the Bioanalyzer (Agilent, Foster City, California, United States) monitoring for ribosomal S28/S18 RNA ratio (acceptable 1.5–2.5), signs of degradation, and DNA contamination. The concentration and the A260/A280 ratio of the samples were measured using a spectrophotometer, the acceptable ratio being 1.8–2.2. We obtained adequate amount of good-quality total RNA from eight MS cases and nine controls for the CD4^−^ cell pool, and for six cases and six controls for the CD4^+^ T lymphocytes. All individuals, from whom we had obtained blood mononuclear cell samples and high-quality RNA, had at least one copy of the MS risk haplotype (C-A), thus we were able to compare the relative expression level of *PRKCA* in individuals with two risk haplotypes to those heterozygous for the risk haplotype.

### Quantitative RT-PCR.

Random hexamers and the TaqMan Gold RT-PCR kit (Applied Biosystems) were used to perform first strand cDNA synthesis starting from 1 μg of total RNA, according to manufacturer's instructions. Real-time PCRs for the quantitation of the *PRKCA* mRNA were carried out using the SYBR-Green assay (Applied Biosystems). Exonic primer couples for *PRKCA,* and for the three genes used in the normalization step (glyceraldehyde-3-phosphate dehydrogenase *[GADPH],* hydroxymethylbilane synthase *[HMBS],* and β-actin *[ACTB]*) were purchased from Proligo (Paris, France), and their sequences are available on request. All reactions were performed in triplicate using the ABI Prism 7900 HT Sequence Detection System, and data were analyzed with the Sequence Detector version 2.0 software (Applied Biosystems).

### Bioinformatic analysis of the genotype and expression data of CEPH individuals.

We obtained the expression and genotype data of CEPH individuals from two recent publications by Monks and collaborators [28] and Hinds and co-workers [29] available in the public databases. Eleven CEPH individuals of European ancestry (NA07019, NA07348, NA07349, NA10831, NA10844, NA10845, NA10853, NA10854, NA10857, NA10860, and NA10861) were used in both studies and thus had both expression and genotype data available. Genotypes for SNPs rs2078153 and rs7215972 for all the 24 CEPH individuals of European ancestry were obtained from http://genome.perlegen.com and used to estimate two-SNP haplotypes [48,49]. SNP rs7215972, which is located between SNPs rs3889477 and rs3890137 (block 7 in Canadian samples, see Figure 2), was utilized instead of rs3890137, since neither of the SNPs in that block were genotyped by Hinds and co-workers [29]. All estimated haplotypes had a probability of 1.0. The expression of *PRKCA* (ID: 10012668758) had been measured in lymphoblast cell lines of 167 CEPH individuals [28], and the data was downloaded from http://www.ncbi.nlm.nih.gov/geo (GSE1726 record). The relative expression level of each gene was given as log_10_ (expression ratio), comparing individual samples to a common pool created from equal portions of RNA from founders within the 15 CEPH families. The average expression level of the *PRKCA* gene was calculated for individuals with two, one, or no copies of the putative disease haplotype (rs2078153–rs3890137: C-A).

## Supporting Information

Table S1 Stage I: SNP Results of the Association Analysis in the Primary Study (29 KB XLS)

Table S2 HHRR Association Results for the Dense Set of 211SNPs in Finnish and Canadian MS Families (58 KB XLS)

Table S3 SNP Haplotypes and the Corresponding *p*-Values (Haploview) Tested in Finnish MS Families (24 KB XLS)

## Abbreviations

CEPH: Centre d'Etude du Polymorphisme Humain
CI: confidence interval
Ct: cycle threshold
HHRR: haplotype-based haplotype relative risk
HLA: human leukocyte antigen
LD: linkage disequilibrium
MS: multiple sclerosis
OR: odds ratio
PKC: protein kinase C
SD: standard deviation
SNP: single nucleotide polymorphism
TDT: transmission disequilibrium test
